# Going, Going, Still There: Using the WebCite Service to Permanently Archive Cited Web Pages

**DOI:** 10.2196/jmir.7.5.e60

**Published:** 2005-12-30

**Authors:** Gunther Eysenbach, Mathieu Trudel

**Affiliations:** ^1^Centre for Global eHealth InnovationTorontoCanada

**Keywords:** Archives, editorial policies, information management standards, digital libraries, periodicals standards, publishing standards, information storage and retrieval, Internet

## Abstract

Scholars are increasingly citing electronic “web references” which are not preserved in libraries or full text archives. WebCite is a new standard for citing web references. To “webcite” a document involves archiving the cited Web page through www.webcitation.org and citing the WebCite permalink instead of (or in addition to) the unstable live Web page. This journal has amended its “instructions for authors” accordingly, asking authors to archive cited Web pages before submitting a manuscript. Almost 200 other journals are already using the system. We discuss the rationale for WebCite, its technology, and how scholars, editors, and publishers can benefit from the service. Citing scholars initiate an archiving process of all cited Web references, ideally before they submit a manuscript. Authors of online documents and websites which are expected to be cited by others can ensure that their work is permanently available by creating an archived copy using WebCite and providing the citation information including the WebCite link on their Web document(s). Editors should ask their authors to cache all cited Web addresses (Uniform Resource Locators, or URLs) “prospectively” before submitting their manuscripts to their journal. Editors and publishers should also instruct their copyeditors to cache cited Web material if the author has not done so already. Finally, WebCite can process publisher submitted “citing articles” (submitted for example as eXtensible Markup Language [XML] documents) to automatically archive all cited Web pages shortly before or on publication. Finally, WebCite can act as a focussed crawler, caching retrospectively references of already published articles. Copyright issues are addressed by honouring respective Internet standards (robot exclusion files, no-cache and no-archive tags). Long-term preservation is ensured by agreements with libraries and digital preservation organizations. The resulting WebCite Index may also have applications for research assessment exercises, being able to measure the impact of Web services and published Web documents through access and Web citation metrics.

## Going, Going, Gone: Lost Internet References as a Growing Problem

Authors of scholarly publications increasingly cite (non-journal) Web pages and other Web-accessible documents in their articles. These cited materials may include for example descriptions of organizations on websites, “grey” research reports which have been published as Web page or Portable Document Format (PDF) files on the Web, online questionnaires, or even data files accessible for example through national statistics websites. As a general rule, published research should be transparent, replicable and falsifiable, and readers should have access to the cited materials, ideally seeing exactly the version authors saw when they cited the material. Yet, cited Web documents are at risk to be changed or even to disappear overnight, being unavailable for future generations of scholars. The unstable nature of Web references is increasingly recognized as a problem within the scientific community, and has been the subject of recent research and science policy discussions [1-8]. It also has been referred to as an issue “calling for an immediate response” by publishers and authors [7]. While services such as the Internet Archive or Google offer archiving (caching) of Internet documents, this is done randomly, does not focus on academic references, and cannot be initiated by authors, editors, or publishers wanting to cache a specific Web reference.

In journals like JMIR, where authors refer to Web services and online information perhaps more often than in other journals, the problem of “link rot” (“broken” links) in the references is particularly pertinent.

## The Solution: Archiving Cited References With WebCite

To prevent “link rot” in scholarly references, JMIR is now among the first journals to adopt a new technology called WebCite (http://www.webcitation.org), which is designed to permanently archive and retrieve cited Internet references. This tool can be used by authors, readers, editors and publishers. It is free of charge for individual scholars (authors and readers), with participating publishers ultimately carrying the operating costs through a membership fee, similar to the CrossRef model, which is a not-for-profit consortium of publishers working on crosslinking between “traditional” journal references which carry a Digital Object Identifier (DOI). The WebCite consortium complements the CrossRef system as it caters to “non-traditional” cited material which does not carry a DOI, and which is therefore typically not permanently preserved in libraries or systems like the LOCKSS (Lots of Copies Keep Stuff Save) project at Stanford University [9].

The following briefly outlines how different groups of stakeholders such as scholars, editors and publishers can use WebCite. JMIR has amended its “Instructions for Authors” accordingly, asking its authors to archive (“cache”) cited URLs preferably before submitting a manuscript [10]. This ensures that peer-reviewers and readers have permanent access to the same version of the cited URL as the author. Thus, the following section entitled “Using WebCite as a ‘Citing Author’” is most pertinent for JMIR authors. If JMIR authors fail to cache cited URLs, archiving will be done later in the article production process, as described below under “Using WebCite as an Editor or Publisher”. However, in these cases the captured version of the cited page may differ from the version the author intended to cite if it has changed between the original access date and the article's processing date, thus authors are urged to prospectively cache cited Web material as early as possible, for example when they create a record in their bibliographic reference management software such as Reference Manager.

### Using WebCite as a Citing Author

On the first level, the caching process can be initiated by the author of a manuscript wishing to cite a Web page (authors should note that it is usually *not* necessary to cache electronic *journal* articles if they have a DOI, as it can be assumed that these are permanently preserved in libraries. However, free articles from e-journals which appear not to be available in libraries, those without an ISSN and/or a DOI should be archived in case they vanish).

To initiate the process, the author goes to webcitation.org and submits the cited URL for archiving before citing it. This process is called to “WebCite®” a Web page or website. The WebCite tool takes a snapshot of the cited Web page and returns a “permalink” (permanent link), which the author should cite in the references section instead of (or in addition to) the unstable live link.

Alternatively, authors may also use a WebCite bookmarklet. A bookmarklet is a small JavaScript program that can be stored as a URL within a bookmark in most popular web browsers, or within hyperlinks on a Web page. The WebCite bookmarklet can be downloaded from the WebCite server and saved to the bookmarks (“favourites”) folder of any Web browser, so that the author can take a snapshot by selecting the bookmarklet whenever he encounters a Web page he might later want to cite.

Other third-party vendors may develop further tools such as browser plug-ins or add-ons to reference management software.

Authors may also cache multiple URLs by initiating a “combing” of a manuscript for URLs (currently this only works for HTML manuscripts). A request to comb the outbound links from a given HTML manuscript leads the WebCite server to present a checklist of outbound links from a manuscript to the user, who can then choose to archive the content of any of the outgoing links. This method is intended to be used during the prepublishing phase of manuscript preparation, in order to capture the content of cited Web pages which the author may have not archived with WebCite during their primary Internet search and writing up of the article. This method is deficient in that the captured version of the cited page may differ from the version the author intended to cite if it has changed between the original access date and the article's processing date. However, in cases where the original author did not include WebCite backed links for their references, this is nonetheless a better approach than simply not caching references at all.

### Using WebCite as a Cited (Web)Author

Authors of online documents and websites which are expected to be cited by others can ensure that their work is permanently available by creating an archived copy using WebCite and providing the citation information including the WebCite link on the Web document(s). They may also put the WebCite bookmarklet as a link on the page(s) they expect to be cited. In the future, cited authors will also be able to retrieve WebCite statistics as an impact measure from webcitation.org.

### Using WebCite as a Reader

Once the page(s) in question have been cached by WebCite, they can be accessed by users and publishers through the webcitation.org server, usually – if implemented by the publisher - just by clicking on a WebCite link next to the reference in question (see references [9] and [10] of this article for examples).

Depending on the information a user has at hand, items cached by WebCite can be queried based on one of three methods: By explicit WebCite ID, by URL and citing article (DOI), or by URL and date.

Retrieval of a cached document by explicit WebCite ID (snapshot ID) is the preferred way to retrieve a specific snapshot. Every item added to the WebCite database (including Web pages, PDF files, and included images or stylesheets) is assigned a unique numeric ID. These IDs are unique, unambiguous and idempotent, and thus represent the ideal way of querying a given resource cached by WebCite. However, the use of this method requires knowing the ID for a given resource, and so it can not be used without premeditation. Upon completion of an archiving request, WebCite sends an email to the user who requested the archiving operation (or, in the case of an FTP uploaded file, to the prearranged technical contact for the DOI prefix of the citing article) containing the WebCite link with the unique ID.

Also possible is retrieval by URL and date. When queried in this manner, WebCite finds all cached versions of the given URL, and sorts them by proximity to the given date. Although this allows for a certain “fudge factor” with timestamps, it also means that these types of queries are inherently ambiguous, and are not guaranteed to be idempotent across queries. As such, these queries are intended to be used when the user has no information in hand other than the URL to query, and possibly the approximate date of the snapshot they would like to see.

The last option, by URL and citing article (identified through its DOI), is the preferred way many publishers may implement a WebCite link. This method allows for publishers which use WebCite as part of their (pre-)publishing workflow to easily create WebCite queries for their cached references with minimal coordination with WebCite before publishing. Publishers submit the citing articles (as XML) shortly before, on or after publication to WebCite, which automatically caches cited URLs. These queries are unambiguous, but are not necessarily guaranteed to be idempotent (the content of the URL may be recached by multiple submissions of a given page for combing).

### Using WebCite as an Editor or Publisher

Journal editors and publishers can use WebCite at three different stages: At the (pre-)submission stage, the copyediting stage, and the publication stage. Ideally, an editor or publisher works with WebCite at all three stages.

On the first level, editors should ask their authors to cache all cited URLs “prospectively” before submitting their manuscripts to the journal, by adding a respective note to their “Instructions for authors” (see this journal [10] for an example).

On the second level, editors and publishers should instruct their copyeditors or “technical” editors (who are in charge of preparing the accepted document for publication) to cache cited Web material if the author has not done so already.

On the third level (a process that is currently tested with JMIR and BioMed Central as early adopters), publishers can submit the raw citing article to WebCite for processing. Ideally, this submission is done via file transfer protocol (FTP), and uses a well defined (preferably XML based) schema for article data. The exact dialect used for this purpose should be agreed on ahead of time by the publisher and WebCite. Currently, WebCite supports (X)HTML documents, NLM Journal Publishing DTD documents, and BioMed Central Article DTD documents. Adding new document types to this list is a straightforward process, and can be undertaken on a publisher by publisher basis by providing WebCite with a document DTD and sample document for testing.

While the first two levels are currently free of charge for publishers, the third level requires that the publisher becomes a member of the WebCite consortium.

A fourth option on how a publisher can use WebCite is retrospective archiving. WebCite also works as a focused crawler, and can – in collaboration with publishers - automatically comb citing articles “retrospectively” for cited URLs. The focused crawler can also be pointed to domains hosting academic journals, which have previously not asked authors to “WebCite” references before submission. Retrospective archiving has the obvious limitation that by the time references are being “WebCite archived” they may have disappeared already.

In a pilot test of the WebCite focussed crawler, WebCite analyzed 280752 references from 8381 articles published in all BioMed Central journals from August 1997 to April 5, 2005. 6627 (2.4%) of these references were “pure” URL citations (i.e. not a URL of a journal article etc.), of which 4919 were unique. 1571 cited an entire domain (i.e. a website as opposed to a specific webpage). 2938 cited a HTML page, 222 a PDF file, and 15 txt/doc files. Obeying a variety of robot-exclusion standards and “no-archive”/”no-cache” metatags or copyright restrictions, WebCite succeeded to archive 3198/4919 (65%) Web pages. 500 were not cached due to robot exclusions, but only 8 had a no-archive and 7 had no-cache restrictions. The remaining Web pages could not be cached because they were already inaccessible or had disappeared.

Due to the limitations of retrospective caching, prospective archiving of cited references by authors or publishers at the time the manuscript is written or published is the preferred way to solve the problem of unstable and dynamically changing Web citations. Since its official launch in October 2005, almost 200 journals are already using WebCite on a routine basis.

## Copyright and Long-Term Preservation Issues

Two of the most frequently asked questions about WebCite concern copyright issues and long-term preservation issues.

First, how does WebCite deal with copyright? Caching and archiving Web pages is widely done (e.g. by Google, Internet Archive etc.), and is not considered a copyright infringement, as long as the copyright owner has the ability to remove the archived material and to opt out. In order to opt out, certain machine readable Internet standards are in use, such as robot exclusion standards, as well as no-cache and no-archive tags, which are all honoured by WebCite. Thus, Web authors of copyrighted material who do not want their work cached or permanently preserved can explicitly exclude it from being archived simply by including these standard tags. In addition to honouring the respective Internet standards, copyright owners of an archived Web page also may request manual removal. In the vast majority of jurisdictions, caching Web pages would also considered “fair use”, in particular because 1) usually only one Web page as part of a larger collection (a website) is quoted, 2) because the webcited document is usually “unpublished” in traditional venues, hence there is no economic impact, with the vast majority of cited authors actually benefiting from the citation, 3) because the webcitation is used in the context of research.

Despite these arguments it has to be acknowledged that copyright legislation and jurisdiction in this area are complex and in a state of flux, and part of a future iteration of WebCite may include a comprehensive licensing management system, allowing to pay royalties to authors of archived material, should they wish so.

Secondly, how can scholars and publishers who opt to use WebCite be sure that the webcitation permalinks themselves will never be broken, that webcitation.org will never disappear? The answer is threefold: First, through the largest possible degree of “openness”: All WebCite code is Open Source, and all documentation is licensed under Creative Commons licenses. Secondly, through collaborations with libraries and consortia interested in preservation of digital material, who may act as a curator, custodian or trustee for the WebCite project. These long-term preservation partners may have agreed to hold backups of the service and to legally assume the domain name, all intellectual property such as trademarks, and the service itself, should for any reason the original WebCite service go out of business. Thirdly, the WebCite consortium will eventually be owned by (or through a membership scheme run by) publishers, who all have a vested interest in keeping the service alive.

## Beyond Archiving: The WebCite Index as a Retrieval and Impact Evaluation Tool

Widespread adoption of the WebCite technology among scholars, editors and publishers will not only solve the problem of inaccessible cited documents, but also open up further possibilities, such as the building – in analogy to the "Science Citation Index" (SCI) – a global “WebCite Index” which has been proposed as early as in 1998 [11]. Such an index can be used as a tool to evaluate electronic publications and websites which are published outside of the traditional peer-reviewed journal publishing route. Currently, websites and electronic documents, even if they are cited heavily, contribute little to a researchers’ career or institutions’ reputation, as they are inadequately captured in the Science Citation Index and in traditional research assessment exercises. Data stored in the WebCite Index can the basis to calculate Web impact measures (the number of times Web documents are cited or accessed provides quality indicators of their importance), activity measures (indicators of research and development activity in the subject areas) and linkage measures (indicators of intellectual linkages between authors/organizations and knowledge linkage between their subject areas).

## Abbreviations

DOI: Digital Object Identifier
DTD: Document Type Definition
HTML: Hypertext Markup Language
LOCKSS: Lots of Copies Keep Stuff Save
NLM: National Library of Medicine
PDF: Portable Document Format
URL: Uniform Resource Locator
XML: eXtensible Markup Language
